# Composite Cardiovascular Outcomes in Patients With Primary Aldosteronism Undergoing Medical Versus Surgical Treatment: A Meta-Analysis

**DOI:** 10.3389/fendo.2021.644260

**Published:** 2021-05-17

**Authors:** Wei-Chieh Huang, Ying-Ying Chen, Yen-Hung Lin, Jeff S. Chueh

**Affiliations:** ^1^ Division of Cardiology, Department of Internal Medicine, Taipei Veterans General Hospital, Taipei, Taiwan; ^2^ School of Medicine, National Yang-Ming Chiao-Tung University, Taipei, Taiwan; ^3^ Division of Nephrology, Department of Internal Medicine, MacKay Memorial Hospital, Taipei, Taiwan; ^4^ Graduate Institute of Clinical Medicine, College of Medicine, National Taiwan University Hospital, Taipei, Taiwan; ^5^ Department of Internal Medicine, National Taiwan University Hospital, Taipei, Taiwan; ^6^ Glickman Urological and Kidney Institute, and Cleveland Clinic Lerner College of Medicine, Cleveland Clinic, Cleveland, OH, United States

**Keywords:** primary aldosteronism, adrenalectomy, mineralocorticoid receptor antagonists, surgical treatment, medical treatment, ROBINS-I

## Abstract

**Background:**

Superior outcomes after surgical treatment over medical treatment for primary aldosteronism (PA) has been reported in small-scale clinical studies, but no solid conclusion has been drawn as results of large randomized trials are lacking.

**Methods:**

We performed a search of PubMed, MEDLINE, Embase and Cochrane Library for randomized or observational studies that investigated cardiovascular outcomes in patients with PA undergoing medical *versus* surgical treatment. Meta-analyses of both composite and individual outcomes were conducted. Risks of bias of the included studies were assessed with Risk Of Bias In Non-randomized Studies of Interventions (ROBINS-I) checklist. Trial sequential analysis (TSA) was performed to control the risk of random errors and assess whether the results in our meta-analysis were conclusive.

**Results:**

A total of 12 studies, including a total of 6148 PA patients, were included in the meta-analysis. The results of meta-analyses demonstrated lower incidence of composite cardiovascular outcomes among PA patients who underwent surgical treatment over medical treatment (odds ratio (OR): 0.49). Surgical treatment also led to less incidence of persistence of hypertension (OR of non-cure hypertension: 0.31). Fewer major cardiovascular events and mortality events were observed (OR: 0.60) after surgical treatment. TSA result showed that the required information size was 2151 and the cumulative Z curve crossed the futility boundary and reached the required information size.

**Conclusion:**

Superior performance of surgical treatment over medical treatment is confirmed with meta-analyses in terms of lower incidences of composite cardiovascular outcomes and non-cure of hypertension. Hence, adrenalectomy could now be concluded as the treatment of choice for lateralized PA.

## Introduction

Primary aldosteronism (PA) was first described in 1955 as hypertension, hypokalemia and overproduction of aldosterone (1). Once thought to be a rare cause of hypertension, PA is now recognized as the most frequent form of secondary hypertension (2) and affects about 5% to 10% of patients with high blood pressure (3). Many studies have shown that PA cases account for around 5% of the general hypertensive population (3), and 11% of hypertension patients are referred to specialized centers (2). Far from being a benign form of hypertension, PA is associated with higher risks of cardiovascular, renal, and metabolic sequelae, including left ventricular hypertrophy, myocardial infarction, atrial fibrillation, stroke, microalbuminuria, osteoporosis, and metabolic syndrome (4–8).

The two most common subtypes of PA are lateralized PA (including aldosterone-producing adenoma (APA), (multiple) aldosterone-producing micronodule(s) (mAPM), or rarely unilateral hyperplasia) and idiopathic hyperaldosteronism (IHA) (9). Targeted treatment with either adrenalectomy (surgery) and mineralocorticoid receptor antagonist (MRA) are the two common comparative, well-documented treatments to improve the outcomes of lateralized PA patients (10, 11). Although adrenalectomy is currently considered as the standard treatment in lateralized PA patients (9, 12, 13), some patients might have residual hypertension and still need anti-hypertensive medications after surgery. The current practice guideline recommends adrenalectomy for lateralized aldosterone excess (9, 12), whereas bilateral disease is treated using MRA (9, 14). However, there are still some patients with lateralized disease who do not undergo surgical intervention. Additionally, the reported discordance rate between a lateralizing adrenal venous sampling (AVS) test and a localizing adrenal CT scan could be as high as 45% (15, 16), which may lead to uncertainty of the final diagnosis and hence management with medical treatment. For these reasons, the relative efficacy of medical *versus* surgical therapy for lateralized PA should be evaluated more clearly.

Previous studies showed similar cardiovascular (10), blood pressure, and hypokalemia (17) outcomes between surgical or medical treatment. However, other studies found adrenalectomy associated with better cure rate of hypertension and hypokalemia (18), improved quality of life (19), as well as lower incidence of cardiovascular adverse outcomes (20). Moreover, one recent study suggested that surgical intervention is superior to medical therapy for lateralized PA due to excess cortisol co-existing in PA patients (21). Therefore, we performed this meta-analysis to elucidate the efficacy between surgical *versus* medical therapy for patients with PA.

## Materials and Methods

### Search Strategy and Selection Criteria

We searched PubMed, MEDLINE, Embase, and Cochrane Library to identify all eligible studies using terms associated with PA. Searches were limited to human studies and patients aged over 18 years old. In order to enhance the number of studies included for screening, broad searching strategies were applied with no language restriction and any relevant reports published after 1985 were included. The key terms used were “hyperaldosteronism,” “hyperaldosertonaemia,” “aldoseteronism,” “aldosteronaemia,” “adrenalectomy (-ies),” “surgery,” “surgical,” “resection,” “mineralocorticoid receptor antagonists,” and “medical treatment.” Searches were done up to Feb 28, 2021.

The searched articles were first evaluated at the title or abstract level and consensus between two independent investigators (WC Huang, YY Chen) or comments from a third reviewer (J Chueh) were sought for any disagreements identified. If the searched articles were potentially relevant, full articles were further retrieved and evaluated as complete reports according to the selection criteria. Studies including prospective study and retrospective studies were selected if the setting of the studies was to evaluate the outcomes (either hypertension-related or clinical events) of PA patients comparing the performance of medical and surgical treatments. Exclusion criteria were non-human setting, duplicate reporting, studies without relevant outcome data, absence of a control group, which referred to the studies presenting the outcomes of either medical or surgical treatments only, or different settings on the intervention and control arms. For example, studies using patients with essential hypertension (EH) as the control group to demonstrate the superior performance of PA patients after either medical or surgical treatment were excluded.

### Data Extraction and Quality Assessment

Two investigators (WC Huang and YY Chen) independently extracted data using a standardized data extraction spreadsheet, with discrepancies resolved by consensus or through a third reviewer (J Chueh). Extracted data included author, journal, year of publication, location of the study group, study design, details of the treatments received by PA patients in both groups, length of follow-up, numbers of participants enrolled in the studies and those who reached the desired end-points of the specific studies. For studies reported in more than one publication, data from the most complete publication was extracted.

The quality of the included studies was evaluated independently by two investigators (WC Huang and YY Chen) using the Risk Of Bias In Non-randomized Studies of Interventions (ROBINS-I) checklist (22), scoring each study for the following 7 domains: “Confounding”, “Selection”, “Classification of intervention”, “Deviation from intervention”, “Missing data”, “Measurement of outcomes” and “Selection of repeated result.” Domains were scored as “No information” (0), “Low” (1—low risk of bias), “Moderate” (2—moderate risk of bias), “Serious” (3—serious risk of bias) and “Critical” (4—critical risk of bias). A study was categorized as high quality if most of the domains were judged to be at low risk of bias.

The pre-defined review protocol was registered at the PROSPERO international prospective register of systematic reviews (http://www.crd.york.ac.uk/PROSPERO, registration number CRD42019119175). The protocol for this trial and supporting CONSORT checklist are available as supporting information; see Checklist S1 and Protocol S1.

### Outcomes of Interests

Efficacy outcomes of interest included: (1) Persistence of hypertension and (2) incidence of major adverse cardiovascular events (MACEs; the composite occurrence of myocardial infarction, stroke, coronary revascularization, or hospitalization because of heart failure) or all-cause mortality. The ‘composite outcome’ [defined as the existence of either (1) or (2)] was considered to overcome the challenges caused by diverse clinical parameters reported in different studies. We considered the presence of either type of outcomes as the incidence of unwanted outcomes after treatments, and the composite outcome was first analyzed to demonstrate the overall effect of the various treatments, and then the treatment efficacy on the individual components was also reported.

### Statistical Analysis

Data were extracted with the use of a standardized data form and any discrepancies were resolved by consensus. In order to compare the differences on the incidence of events between the 2 treatment groups, pooled odds ratios (ORs) with corresponding 95% CI using the Mantel-Haenszel random-effects models was constructed with the aid of RevMan 5.3 (The Cochran Collaboration, The Nordic Cochrane Centre, Copenhagen, Denmark). The assigned weights in the random-effects model considered the variances of both within-studies and the between-studies.

The heterogeneity between the studies was evaluated using the Cochran Q test and the null hypothesis of statistical homogeneity was rejected if p values found were less than 0.10. The extent of heterogeneity was further categorized based on the value of I^2^: mild (less than 30%), moderate (30–50%) and substantial (>50%). Graphical inspection on generated funnel plots was applied to detect publication bias.

### Trial Sequential Analysis

Trial sequential analysis (TSA) was performed with the tool developed by the Copenhagen Trial Unit, Centre for Clinical Intervention Research, Denmark. In order to see the different temporal traceries or clinical outcome of current targeted treatments, e.g. adrenalectomy or MRA, we applied TSA in enrolled 12 studies to control the risk of random errors and simultaneously minimize the chance of having type I errors (5%) due to random error from the included studies with small sample size, potential bias and low methodological quality, together with the influence of the inclusion of studies with comparatively large sample sizes. We used a random effects model to construct the cumulative Z curve and used an anticipated relative risk reduction (RRR) of 15.0% with a power of 90% to calculate the required information size to detect or reject an intervention effect. The incidence in control arm was adjusted to 37.5% in this study. The conventional nonsuperiority boundaries were calculated assuming significance levels of .05 and .05, and a power of 90%. The a-spending boundaries were also calculated using significance levels of .01 and .05 and the O’Brien-Fleming multiple testing procedure (23). When the cumulative Z curve crossed the trial sequential monitoring boundary or entered the futility area, a sufficient level of evidence for accepting or rejecting the anticipated intervention effect may have been reached, and no further studies were needed. If the Z curve did not cross any of the boundaries, and the required information size had not been reached, evidence to reach a conclusion was insufficient, and more studies would be required (24). We used TSA version 0.9.5.5 (reviewed in November 2016) b software was used for these analyses the cumulative effect of randomized trials on mortality.

## Results

### Included Studies

**Figure 1:**
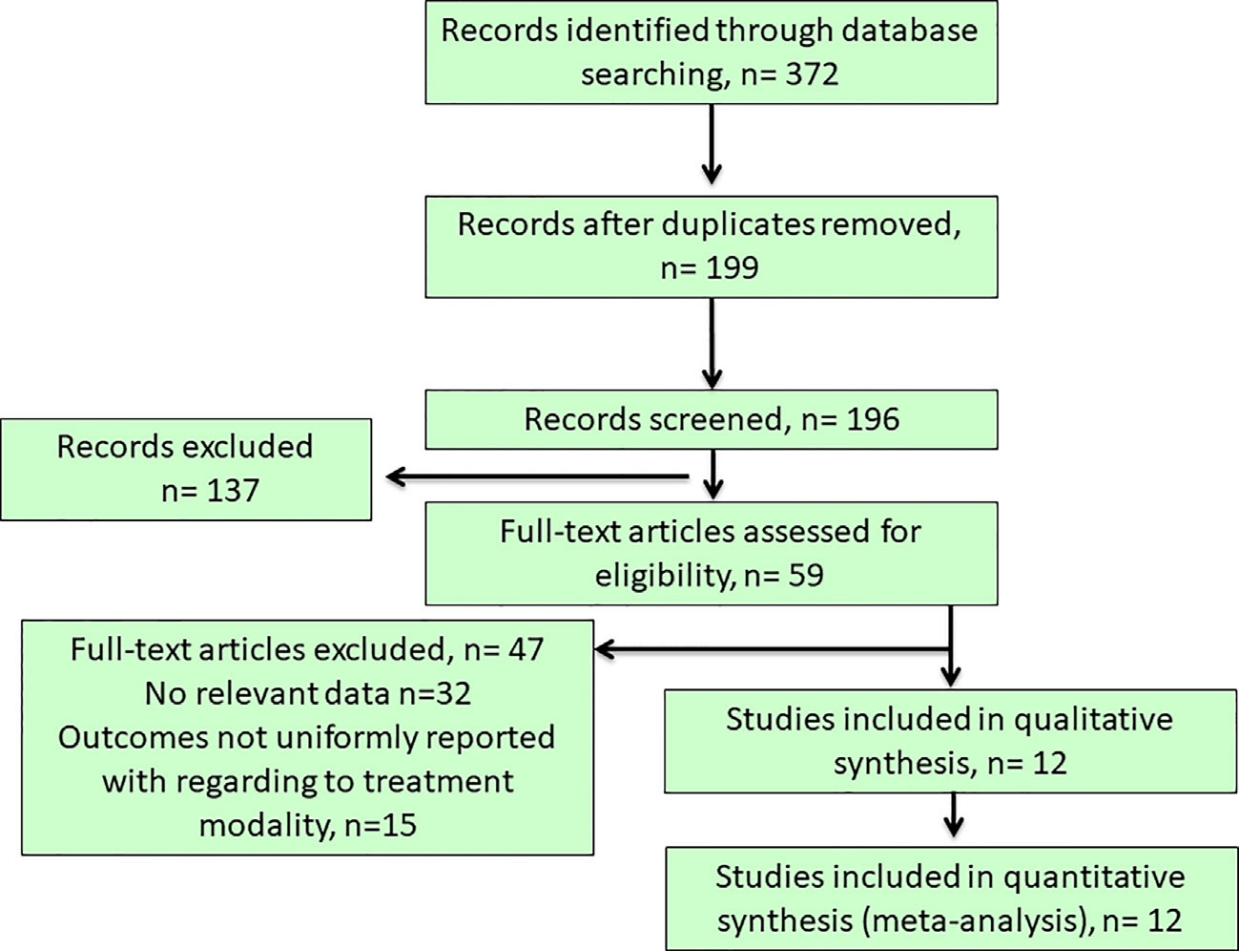
Flow chart of literature search and study selection. Our initial literature search yielded a total of 372 articles from the selected databases and after the removal of duplicated and irrelevant records, 59 articles with full-text articles were further reviewed. Among them, 32 did not show relevant data, while other 15 articles reported outcomes non-uniformly with regard to the target treatments. Finally, 12 studies was included in this study.

Our initial literature search yielded a total of 372 articles from the selected databases and hand-searching references through the applied searching strategy and they were further evaluated for eligibility at title or abstract level (**Figure 1**). After the removal of duplicated and irrelevant records, 59 articles with full-text articles were further reviewed. Among them, 32 did not show relevant data, while other 15 articles reported outcomes non-uniformly with regard to the target treatments. Finally, 12 studies (6, 10, 18, 20, 25–32) were included in this meta-analysis (**Figure 1**), resulting in a total of 6148 PA patients who received either surgical (35.98%) or medical treatment (64.02%). The descriptive summary for each included study was recapitulated in **Table 1**. Types of studies included prospective studies (n = 6) and retrospective studies (n = 6). Moreover, our previous study had a heavy weight in this meta-analysis as even the analysis of the outcomes to either surgical or medical treatment was carried out at the sub-group analysis level, its large sample size gave the advantage to maintain study quality, although a separate EH database was used as a control group which underwent standardized PA screening evaluation to exclude the possibility of subclinical PA (30).

**Table 1:** Characteristics of the trials included in the meta-analysis.

Reference	Study nature	Relevant outcomes	Mean duration of follow-up (months)	Number of patients	
				Surgical	Medical
Bernini et al. (20)	Prospective clinical trial	Hypertension-related	Surgical: 31.5 Medical: 32.1	19	41
Catena et al. (10)	Prospective clinical trial	Hypertension-related	76.8	24	30
Catena et al. (27)	Prospective clinical trial	Composite cardiovascular end-points	60.0 (selected)	24	30
Giacchetti et al. (29)	Prospective clinical trial	Hypertension-related	34.4	25	36
Kline et al. (18)	Retrospective study	Hypertension-related	Surgical: 6.5 Medical 13.4	38	39
Miyake et al. (25)	Retrospective study	Hypertension-related	60.0 (max)	755	800
Mulatero et al. (6)	Retrospective study	Composite cardiovascular end-points	144.0 (median)	57	213
Park et al. (31)	Retrospective study	Hypertension-related	Surgical: 45.6 Medical: 55.2	206	63
Rossi et al. (28)	Prospective clinical trial	Hypertension-related	36.0 (median)	110	70
Wu et al. (30)	Retrospective study	Composite cardiovascular end-points or all-cause mortality	69.0	846	2516
Zacharieva et al. (26)	Prospective clinical trial	Hypertension-related	Surgical: 3.0 Medical: 3.0-6.0	22	30
Puar et al. (32)	Retrospective study	Composite cardiovascular end-points	68.4	86	68

### Quality Assessment

The result of ROBINS-I tool analysis showed that the overall risk of bias of the majority of the included studies was from low (1) to moderate (2) (**Table 2**). Two of the included retrospective studies failed to report the handling methods of missing data (18, 30), which might introduce bias, hence their overall risks of the bias were relatively higher. Due to the restriction of small sample size of the included clinical studies, most of the included studies showed limitations on adjusting the outcome measures with potential confounding variables (domain 1), and in two of the included studies serious (25, 30) (3) to critical (4) bias in this domain was further pointed out. In addition, the risk of bias due to selection of reported results (domain 7) among the included studies reached moderate level.

**Table 2:** Range of overall assessment by study and bias domains.

	Domain 1: confounding	Domain 2: selection	Domain 3: classification of intervention	Domain 4: deviation from interventions	Domain 5: missing data	Domain 6: measurement of outcomes	Domain 7: selection of reported result	ROBINS-I overall
Bernini et al. (20)	2-3	1	1	1-2	1	2-3	2	1-2 Low – Moderate
Catena et al. (10)	3	1	1	1-2	1	2-3	2	1-2 Low – Moderate
Catena et al. (27)	2	1	1	1-2	1	2-3	2-3	1-2 Low – Moderate
Giacchetti et al. (29)	3	1	1	1-2	1	2-3	2	1-2 Low – Moderate
Kline et al. (18)	3-4	1	1	1-2	2	2-3	2	2-3 Moderate – Serious
Miyake et al. (25)	2	1	1	1-2	3-4	2-3	2	2-3 Moderate – Serious
Mulatero et al. (6)	2-3	1	1	1-2	1	2-3	3	3 Serious
Park et al. (31)	2	1	1	1-2	1	2-3	2-3	1-2 Low – Moderate
Rossi et al. (28)	3-4	1	1	1-2	1	2-3	2	2 Moderate
Wu et al. (30)	2	1	1	1-2	3-4	2-3	2-3	3 Serious
Zacharieva et al. (26)	3-4	1	1	1-2	1	2-3	2	2 Moderate
Puar et al. (32)	2	1	1	1-2	1	2-3	2	1-2 Low – Moderate

Risk of bias assessment: 0 No information; 1 Low; 2 Moderate; 3 Serious; 4 Critical

### Trial Sequential Analysis

**Figure 2:**
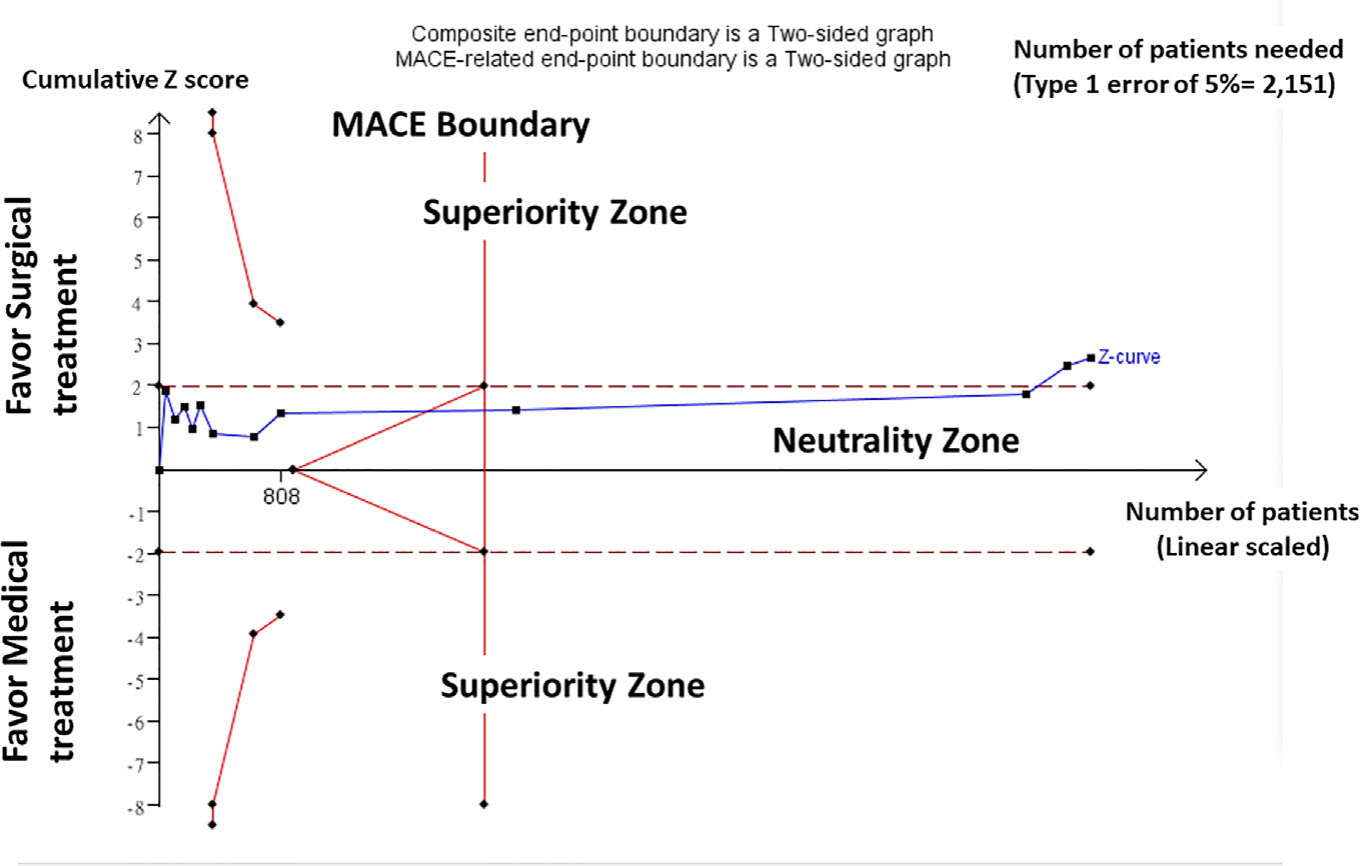
Trial Sequential Analysis (TSA). Result of TSA showed homogeneity of results was stable and around 2151 patients would be needed to reach a stopping boundary of superiority. The Z-curve was parallel to superior boundary, but crossed the neutrality boundary including all trials. Trials of patients yielded enough information size to conclude that the current evidence reached preliminary conclusion for supporting the superior outcome of surgical treatment over medical treatment.

In a TSA on the composite negative outcomes including both non-cure of hypertension and incidence of adverse cardiovascular events and all-cause mortality, random-effects model (DL) was applied in the TSA for all 12 included trials. TSA performed using a significance level of 0.05 and considering that homogeneity of results was stable showed that around 2151 patients would be needed to reach a stopping boundary of superiority. However, the Z-curve was parallel to superior boundary, but crossed the neutrality boundary including all trials. In our TSA, we included trials of patients, which yielded enough information size to conclude that the current evidence reached preliminary conclusion for supporting the superior outcome of surgical treatment over medical treatment (**Figure 2**).

### Primary Outcome: Incidence of Outcomes

**Figure 3:**
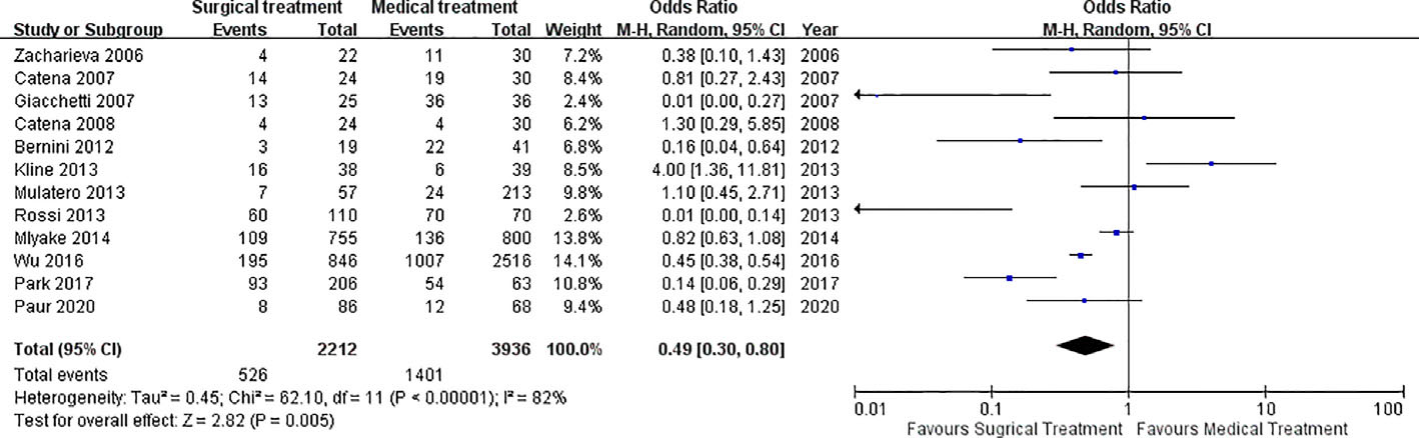
Forest plot of the OR of primary outcome. Forest plot of the OR of primary outcome (composite outcomes) in patients with primary aldosteronism under medical treatment or surgical treatment. Central squares of each horizontal line represent the OR for each study. Horizontal lines indicate the range of the 95% CI and the vertical line indicates an OR of 1.0 (which indicates no differences in the odds ratio between medical treatment or surgical treatment). OR, odds ratio.

Among the 12 included studies, 8 of them reported the persistence of hypertension as primary outcome of the studies, while the other 4 studies reported the occurrence of MACEs or all-cause mortality as the primary outcome of the studies. Superior performance of surgical treatment over medical treatment was reported in 4 included studies, in regard to cure of hypertension and event-free of MACEs or all-cause mortality. Conversely, two included studies reported superior performance of medical treatment for PA patients over surgical treatment, in term of lower incidence of persistence of hypertension. The remaining 6 studies failed to give a definite conclusion on whether the medical or the surgical treatment was better for PA patients. Overall, when the composite outcomes were considered with the use of random effects model, the significance of better outcome in adrenalectomy (surgery) group was observed with the odds ratio [(OR) (OR: 0.49 (95% CI: 0.30-0.80, p = 0.005] (**Figure 3**). However, there was a significant heterogeneity among the enrolled studies [I (2) = 82%, p < 0.00001]. In the random effect models of meta-analysis on the composite outcomes, symmetry in the funnel plots was observed and this implied low publication bias among the included studies, but outliers also existed due to the diversity on the scales of the included studies (**Figure S1** and **Supplemental File**).

When the cure of hypertension after targeted treatment was considered, superior performance of the adrenalectomy was demonstrated in the random effects model (OR: 0.31, 95% CI: 0.11 – 0.85, p=0.020) (**Figure S2** and **Supplemental File**). For significant clinical events including both mortality or MACEs, surgical treatment led to lower incidence of outcomes when compared with that of medical treatment (OR: 0.60, 95% CI: 0.36 – 1.01, p = 0.05) (**Figure S3** and **Supplemental File**).

## Discussion

### Main Findings

In this meta-analysis of 12 studies that included 6148 patients with PA, both composite and individual outcomes after surgical *versus* medical treatment have been reviewed systematically. Among 8 out of these 12 included studies were related to cure of hypertension of the enrolled PA patients at follow-up period after targeted treatments, and the integrated result suggested that adrenalectomy has a high possibility of achieving cure of hypertension. This result echoed with the findings of a retrospective study with large sample sizes (25). In addition, our finding suggested the significance of superior outcomes of the adrenalectomy in the meta-analysis of composite outcomes, including MACEs, all-cause mortality and persistence of hypertension among the PA patients and, hence, adrenalectomy might be considered in the treatment of choice for uPA.

In the Primary Aldosteronism Surgical Outcome (PASO) study (33), which included relevant data of 705 PA patients who underwent adrenalectomy, the prevalence rate of complete (“normal blood pressure without the aid of antihypertensive medication”) and partial (“the same blood pressure with less antihypertensive medication or a reduction in blood pressure with either the same amount or less antihypertensive medication”) clinical success was summed up as high as 84.2%, together with 93.9% biochemical success, which refers to “correction of hypokalemia and normalization of aldosterone to renin ratio (ARR)”. In this meta-analysis, the percentage of PA patients with hypertension cured after surgical treatment was higher than that with medical treatment (73.98% vs 68.08%). This remarkable finding suggests that adrenalectomy is an effective treatment for lateralized PA patients. In addition, a systematic review on comparing the outcomes of PA patients after surgical *versus* medical treatment also reported reduced usage of antihypertensive medications, improved quality of life and potential lower all-cause mortality after surgical treatment (34). Additionally, a number of large case series have confirmed that surgical treatment (unilateral adrenalectomy) of lateralized PA is safe with low morbidity. No major differences in cure rate between various surgical techniques were seen, but some authors have identified predictive characteristics for cure (35–37). Reported complication rates of adrenalectomy ranged between 2 and 10%, with the highest rate occurred in an early series (38).

Long-term treatment with the MRA, including both spironolactone and/or eplerenone, was reported to have 5 – 8% remission rate of PA, this additional beneficial effect made the medical treatment an alternative of surgical treatment, especially for those patients with bilateral PA (12). However, based on our findings in this meta-analysis, the efficacy of the medical treatment for lateralized PA patients was not comparable to that of surgery. One of the potential causes is that the mineralocorticoid receptors, with high bonding affinity with both mineralocorticoids and glucocorticoids, were reported to be involved in the maturation of pre-adipocytes to adipocytes (34), which could be associated with the higher prevalence of hyperglycemia among PA patients over patients with EH (39). Since there is no clear evidence that MRA could cause better control on both glucose and lipid levels in PA patients, the efficacy of medical treatment becomes questionable in regard to its influence on metabolic syndrome. Meanwhile, many case reports and case series reported excess glucocorticoid secretion among PA patients (40, 41). MRA treatment may induce a further increase in aldosterone and subsequently trigger a vicious cycle that leads to an insufficient effect of prescribed MRA on blocking MRs which are activated by high plasma aldosterone levels. Arlt et al. further reported that excess glucocorticoid secretion could be associated with several risk factors which might cause adverse metabolic events among PA patients, and additional glucocorticoid-opposing treatment should be provided for PA patients in addition to MRA treatment, in order to reduce the potential risks of having cardiovascular diseases and other comorbidities (21). Although, there were many studies supporting that glycometabolic abnormalities when excess aldosterone is not removed with use of MRAs, Catena et al. (42) stated that in primary aldosteronism, normal sensitivity to insulin is rapidly restored after treatment with either adrenalectomy or aldosterone antagonists, whereas no further change of glucose metabolism parameters occurs during the long-term follow-up. In recent studies, adrenalectomy in patients with APA was found to decrease glucocorticoid secretion, reverse osteoporosis, attenuate adverse metabolic risks and improve the quality of life (13, 21, 43, 44); all of which could be attributed to decreased glucocorticoid levels after adrenalectomy, in addition to correction of mineralocorticoid excess. Such finding further adds insights on our results, and it could at least in part explain the relatively inferior performance of medical treatment over adrenalectomy, although the adoption of medical treatment itself is not the direct cause of higher incidence of cardiovascular comorbidities.

Patients treated medically need more anti-hypertensive drugs (34), and require a longer follow-up and more clinical visits at specialized referral center than those treated surgically. Similarly, the incidence of cardiovascular events during the follow-up period was greater in IHA patients than that in EH controls, although there were no differences in their BP levels (6). It implicates that aldosterone may play a detrimental role independent of its effect on blood pressure. MRA may also induce an increase in aldosterone and subsequently trigger a vicious cycle that leads to an insufficient effect of prescribed MRA on blocking MRs activated by high plasma aldosterone levels. There are also concerns about MRA prescription, such as medication compliance with inadequate dose, probably partially due to frequent occurrences of dose-dependent side effects, especially gynecomastia or dysmenorrhea, and a subsequent decrease in efficacy (30).

### Strengths and Limitations

Recommendations on treatment are hampered by the lack of systematic reporting of clearly defined outcomes and randomized clinical trials (RCTs). The present data supported surgical resection of lateralized PA disease, which can be performed with low morbidity. Some researchers (34) have attempted to answer this question before, but only qualitative comparisons between these targeted treatments were made due to diverse and heterogeneous clinical outcomes reported from clinical studies and lack of RCTs on this research topic, which is considered as critical evidence for constructing such a meta-analysis. In this meta-analysis, composite outcome approach expanded the scope of the included studies successfully and achieved a sufficiently large sample size to embrace the diversity on the settings of clinical studies and overcome the limitations of small-scale clinical studies. With sufficient sample size, a more concrete conclusion on the superior performance of surgical over medical treatment for lateralized PA patients could be drawn with good-quality quantitative evidence.

In addition, ROBINS-I was adopted in this meta-analysis to assess the risks of bias among the included studies. ROBINS-I was developed by Sterne et al. and it was designed to evaluate the potential risk of bias in the non-randomized studies of the effects of interventions (22). Through answering the signaling questions included in the checklist, the risks of bias for 7 domains of each included study were first reviewed and subsequently, overall risk of bias of the entire study was then concluded. The adoption of ROBINS-I in this study provided a comprehensive evaluation on the quality of the included studies and revealed that most of the included clinical trials were with low to moderate risks of bias while the retrospective studies possessed higher risks of bias.

We acknowledge several limitations of our study. First, there was no adjustment on the results of the meta-analyses according to other influential variables such as patient demographics. Furthermore, lack of the dosage of MRA, types of medication or targets in the medical treatment might further influence the outcomes of PA patients. Second, the drawback of composite outcome approach introduced large heterogeneity within the meta-analysis, especially since there was significant difference on sample sizes of the included studies. Furthermore, the pathology of APA and IHA differ, and these differences lead to diversity in clinical outcomes after the same treatment (45). Because surgery is not an option for patients with IHA (46), our findings are clinically relevant only to patients with APA/lateralized PA. Especially, it is important to accurately diagnosis aldosterone-producing microadenoma in which has better rate of cure of hypertension than macroadenoma and is only detected by AVS (47). Finally, due to so small enrolled patients and the different temporal traceries or clinical outcome of current targeted treatments, e.g. adrenalectomy or MRA, we performed trial sequential analysis (TSA) and in our TSA, was obvious that the included trials over the years (from 2006 to 2020) showed certain extent of consensus on demonstrating constant performance of adrenalectomy under the adjustment of TSA.

## Conclusion

In conclusion, superior performance of surgical treatment over medical treatment for patients with lateralized PA has been confirmed with this meta-analysis approach on both composite and individual clinical cardiovascular outcomes. Adrenalectomy should be concluded as the treatment of choice for the management of lateralized PA patients, especially in regard to attenuating unwanted cardiovascular events.

## Data Availability Statement

The original contributions presented in the study are included in the article/**Supplementary Material**. Further inquiries can be directed to the corresponding author.

## Author Contributions

W-CH contributed to study design, data collection, statistical analysis, data interpretation, and drafting of the manuscript. W-CH and Y-YC contributed to study design, data collection, data interpretation, and critical review of the manuscript. Y-YC and Y-HL contributed to statistical analysis and critical review of the manuscript. JC contributed to data interpretation and critical review of the manuscript. JC is the guarantor of this work and, as such, had full access to all the data in the study and takes responsibility for the integrity of the data and the accuracy of the data analysis. All authors contributed to the article and approved the submitted version.

## Conflict of Interest

The authors declare that the research was conducted in the absence of any commercial or financial relationships that could be construed as a potential conflict of interest.
