# Supplementary material for: Composite Cardiovascular Outcomes in Patients With Primary Aldosteronism Undergoing Medical Versus Surgical Treatment: A Meta-Analysis
Source: Front Endocrinol (Lausanne). 2021 May 17;12:644260. doi: 10.3389/fendo.2021.644260 (PMC8165438; doi:10.3389/fendo.2021.644260)
Supplement: Supplementary file 1 [file DataSheet_1.docx]

Supplementary Information

sFigure 1 Funnel plot of primary outcome. (broken lines show overall effect and 95% confidence intervals)


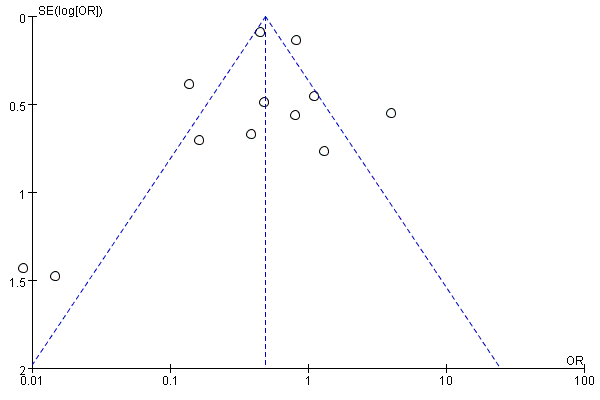


sFigure 2: Subgroup analysis: persistence of HT with the application of random effects models


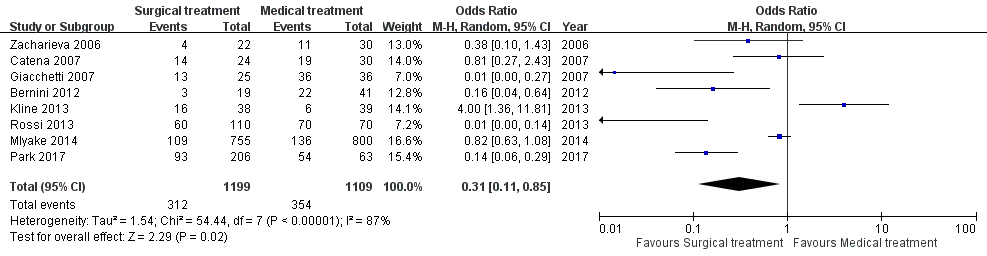


sFigure 3 Subgroup analysis: incidence of cardiovascular outcomes or all-cause mortality with the application of random effects models


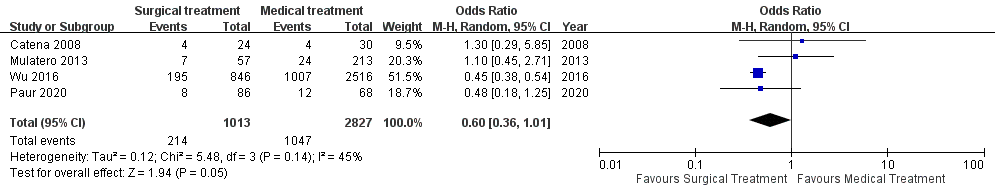


Changes in systolic blood pressure (SBP) and diastolic blood pressure (DBP) of PA patients at the end of follow-up periods of the included trials, as the potential secondary outcomes were compared between 2 groups. 5 out of 12 trials have reported mean SBP and DBP of the enrolled PA patients at both baseline and the end of the follow-up period. Among these 5 trials, SBP and DBP of the enrolled PA patients (n = 501; medical treatment = 234 and surgical treatment = 267) received either treatment were significantly reduced after the treatments (p < 0.050), but none of these 5 trials evaluated the impact of different treatments on the change of BP. Overall, when the random effect model was applied, no significant difference between treatments was observed on SBP and DBP lowering effect at the end of the follow-up (SBP: -0.55, 95% CI: -3.50 – 2.39, p = 0.710; DBP: -2.36, 95% CI: -5.70 – 0.98, p = 0.170) (figure e1 & e2).

Supplementary figure 1 (e1):

Secondary outcome: changes on SBP with the application of random effect model


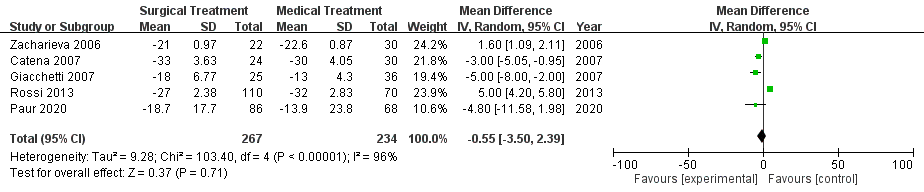


Supplementary figure 2 (e2):

Secondary outcome: changes on DBP with the application of random effect model


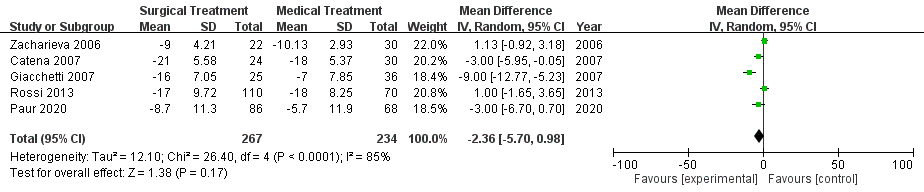


Supplementary Table 1: the criteria adopted for each of the included studies for PA confirmation and PA subtype diagnosis

| Reference | Case detection | Case confirmation | Subtype classification |
| --- | --- | --- | --- |
|  |  |  |  |
| Bernini, 2012 | evaluation of the ALD (ng/dl)/plasma renin activity (PRA) (ng/ml/h) ratio on at least 2 occasions (ALD/PRA ratio over 69 [cut-off of normality in our laboratory, as recently published] and plasma ALD>15 ng/dl | captopril test (ALD values remained >15 ng/dl )  saline loading test ALD values remained >7 ng/dl) | high-resolution computed tomography scan (CT)  adrenal venous sampling (AVS) (62/66) |
| Catena, 2007 | increased plasma aldosterone: active renin ratio (≥20) | intravenous saline load | high-resolution computed tomography scan, followed by selective adrenal vein sampling (n=14) and/or adrenal scintigraphy with iodocholesterol (n=47) |
| Catena, 2008 | increased plasma aldosterone to active renin ratio ≥20 | intravenous saline load | high-resolution CT followed by selective adrenal vein sampling (n=14) or adrenal scintigraphy (n=47) performed using iodocholesterol I 131 with dexamethasone suppression |
| Giacchetti, 2007 | Before 2001  After 2001: ARR (cut-off level: 40 [(ng/dl) per (ng/ml per h)]+Aldo >15 ng/dL | intravenous 4-h saline infusion: post-test aldosterone value was greater than 7 ng/dl | Computerized tomographic (CT) scan +/- MRI  AVS (n=12) |
| Kline, 2013 | measurement of ARR: ARR greater than 550 (aldosterone in pmol/l and renin activity in ng/ml/hour; equivalent to cutoff of 15 when aldosterone expressed in ng/dL)  Anti-HTN agents are not routinely stopped with the exception of mineralocorticoid antagonists |  | Adrenal imaging and AVS (AVS is only performed on PA patients who are considered to be surgical candidates, PA patients with complex, serious medical comorbidities are excluded from the analysis) |
| Miyake, 2014 | complete a simple questionnaire on the number of inpatients and  outpatients with one of the eight disorders of adrenal hormones, including PA | Not mention | Not mention |
| Mulatero, 2013 | positive ARR was 40 (nanograms per deciliter per nanogram per milliliter per 1 hour per 1-1) (50 before 2001) together with an aldosterone level greater than 15 ng/dL | saline infusion test: posttest aldosterone levels were greater than 5 ng/dL | CT  AVS (237 pts success, 88%) |
| Park, 2017 | increased plasma aldosterone to renin activity ratio (≥20) and plasma aldosterone levels of >15 ng/dL | saline infusion test | adrenal gland computed tomography and adrenal venous sampling |
| Rossi, 2013 | ARR (at baseline and after captopril) | saline infusion test | High-resolution computed tomography (CT) scan +/- MRI  AVS (without ACTH stimulation) (43/126)  Dexamethasone-suppressed adrenocortical scintigraphy (when AVS unavailavble) |
| Wu, 2016 | NHI database | NHI database | NHI database |
| Zacharieva, 2006 | ARR >35 | Not performed | CT |
| Paur 2020 | Aldosterone–renin ratio (ARR) | Using either the intravenous saline–infusion test (post–saline aldosterone >5ng/dL) or oral salt loading | Computed topography (CT)  AVS |
